# Atomic insights into the effects of pathological mutants through the disruption of hydrophobic core in the prion protein

**DOI:** 10.1038/s41598-019-55661-2

**Published:** 2019-12-16

**Authors:** Juhwan Lee, Iksoo Chang, Wookyung Yu

**Affiliations:** 1 Center for Proteome Biophysics, DGIST, Daegu, 42988 Korea; 2 Department of Emerging Material Sciences, DGIST, Daegu, 42988 Korea; 3 Core Protein Resources Center, DGIST, Daegu, 42988 Korea; 4 Supercomputing Bigdata Center, DGIST, Daegu, 42988 Korea; 5 Department of Brain and Cognitive Sciences, DGIST, Daegu, 42988 Korea

**Keywords:** Computational biophysics, Molecular biophysics, Biological physics

## Abstract

Destabilization of prion protein induces a conformational change from normal prion protein (PrP^C^) to abnormal prion protein (PrP^SC^). Hydrophobic interaction is the main driving force for protein folding, and critically affects the stability and solvability. To examine the importance of the hydrophobic core in the PrP, we chose six amino acids (V176, V180, T183, V210, I215, and Y218) that make up the hydrophobic core at the middle of the H2-H3 bundle. A few pathological mutants of these amino acids have been reported, such as V176G, V180I, T183A, V210I, I215V, and Y218N. We focused on how these pathologic mutations affect the hydrophobic core and thermostability of PrP. For this, we ran a temperature-based replica-exchange molecular dynamics (T-REMD) simulation, with a cumulative simulation time of 28 μs, for extensive ensemble sampling. From the T-REMD ensemble, we calculated the protein folding free energy difference between wild-type and mutant PrP using the thermodynamic integration (TI) method. Our results showed that pathological mutants V176G, T183A, I215V, and Y218N decrease the PrP stability. At the atomic level, we examined the change in pair-wise hydrophobic interactions from valine-valine to valine-isoleucine (and vice versa), which is induced by mutation V180I, V210I (I215V) at the 180^th^–210^th^ (176^th^–215^th^) pair. Finally, we investigated the importance of the π-stacking between Y218 and F175.

## Introduction

Transmissible spongiform encephalopathies are neurodegenerative diseases caused by the misfolding and aggregation of prion protein (PrP)^1–3^. PrP undergoes a conformational change from normal cellular isoform, PrP^C^, to a misfolded isoform of prion proteins, PrP^SC^, due to infections, sporadic disorders, or genetic factors^4^. In particular, 10%–15% of prion diseases are related to genetic mutations, and many pathogenic mutants of PrP have been reported^5^. Therefore, thermodynamic destabilization, which leads to misfolding and aggregation of PrP, has been studied for such pathogenic mutants^6–8^.

PrP is anchored to the neuronal cell membrane with C-terminal-linked glycosylphosphatidylinositol (GPI). In addition, PrP has two N-glycosylation sites, at N181 and N197 (Fig. S1). The locations of the two glycosylation motifs in PrP are the middle of the second (H2) and third (H3) helix bundles (N181-I182-T186; first motif), and the loop region between H2 and H3 (N197-F198-T199; second motif). Glycosylation starts with the synthesis of the glycoprotein, and glycan, which is covalently bonded to the protein, increases the structural stability and solubility, in addition to determining the cellular localization of PrP^9–11^. Many previous studies on pathogenic mutant T183A have discussed the role of first N-glycosylation motif (N181-I182-T186). The patients with this mutation typically suffer from dementia, cerebral atrophy, and hypometabolism^12^. The V180I mutant, which is close to the first glycosylation motif, is implicated in the Creutzfeldt-Jakob disease (CJD)^13^. Both pathological mutants affect membrane anchoring^14^. Interestingly, a previous study about GPI anchoring reported that changes in the packing of the hydrophobic core prevent the anchoring of GPI to the neuronal cell membrane^15^. T183A and V180I are also located on the hydrophobic core. *In-silico* studies on T183A and V180I have reported that these mutants could affect the structural stability of the hydrophobic core^16^. In many protein systems, hydrophobic interaction is the main driving force for protein folding as well as a strong determinant of thermostability^17^. Many pathological mutants located in the hydrophobic core have been reported in PrP. For instance, V176G has been reported in Gerstmann-Sträussler-Scheinker disease (GSS) patients^18^. V210I mutants was found to be associated with Creutzfeldt-Jakob disease (CJD)^19^. I215V has been implicated in pathogenic Alzheimer’s disease (AD) and CJD^20^. Y218N has been found in GSS patients^21^. In this study, we focus on the thermodynamic stability of these pathological mutants, which are associated with the components of the hydrophobic core.

To compare the thermostability between wild-type and mutant proteins, many computational methods have been developed, which calculate the free energy difference (ΔΔG = ΔG_Wild_ – ΔG_Mutant_, Fig. S2) associated with a single point mutation. However, there are two main hurdles to enhancing the accuracy of these methods. One is the protein structure search problem in the three-dimensional conformational space. The protein structure has a dynamic motion, traveling the local minima in the conformational space. Therefore, the structural stability needs to be calculated for every allowed conformation at a given temperature. The second problem is the scoring of the energy function. Force fields are usually made from physical-based potentials (PBP), statistical knowledge-based potentials (KBP), or a hybrid of the two. PBP consider physical forces between atoms, and CC/PBSA^22^ and EGAD^23^ are examples of PBP-based programs. KBP are based on statistical analysis extracted from known protein structures, and FoldX^24^ is a KBP-based program. Rosetta^25^ uses a hybrid scoring energy function based on both PBP and KBP. A previous review about these programs reported that all computational methods predict a correct trend, but the correlation coefficients between the calculated and experimental change in protein stability (ΔΔG) range from 0.26 to 0.59^26^. These results indicate the need for developing more accurate methods for protein stability calculation. One of the molecular dynamics (MD) simulation protocols, the thermodynamic integration (TI) method, with an AMBER force filed, has been recently proposed for the calculation of protein stability, and it shows a great agreement with experimental data (correlation coefficient = 0.86)^27^.

To overcome the two abovementioned hurdles to enhancing accuracy, we ran the temperature-based replica exchange molecular dynamics (T-REMD) simulation with a cumulative simulation time of 28 μs (14 replicas and 2 μs for each replica) for wild-type PrP. T-REMD allows extensive conformational ensemble sampling across the local minima, with proteins traveling the various system temperatures. We chose 2,100 snapshots from T-REMD for the initial structure of TI calculation, and we performed TI simulation with a cumulative simulation time of 113.4 μs. For the TI calculation, Hamiltonian is related to λ as:

$${H}_{\lambda }={H}_{wt}+\lambda ({H}_{mut}-{H}_{wt})$$

when the λ values are changed from 0 to 1, the protein system evolves from wild-type to mutant protein, and dummy atoms to real atoms. During the TI calculation, we run the MD simulation with a hybrid state (Fig. S2) that system coexists both wild-type and mutant amino acid simultaneously at the same position with a different proportion depending on the λ value. For example, the system is a hybrid structure of 30% of the mutant structure and 70% of wild structure when λ is 0.3. This method is useful to tracks the interaction partners of wild-type and mutant amino acids at the same time. This sequential change of the λ value allowed us to compare the stability between wild-type and mutant PrP. Thus, we calculated the protein folding free energy differences of six pathogenic mutants using thermodynamic cycle (Fig. S2) with a cumulative simulation time of 113.4 μs, and compared them with experimental results.

## Materials and Methods

### Temperature-based replica-exchange molecular dynamics (T-REMD)

Extensive structure ensemble sampling is essential for the accurate prediction of protein thermostability. The energy barrier across the local minima acts as a main obstacle when a protein traverses the energy landscape during MD simulation. In this study, we used the T-REMD method to overcome energy barriers and obtain extensive structure ensemble. T-REMD, the most popular modern method for this purpose, is used to map the free energy landscape based on system temperature exchanges. Close temperature intervals (~4.3 K) between the replicas were used to facilitate the exchange ratio^28^. The average exchange rate is 1.2%, and exchanges are attempted every 0.1 ns (replicas have 3,332 temperature exchange during T-REMD simulation). Human PrP with residues 125–228 (PDB code: 1QLX)^1^ and the TIP3 water model^29^ were used for the initial structure T-REMD simulation. AMBER18 simulation package, with the ff14SB force field, was used for all simulations^30^. The ff14SB force field increased the accuracy of helix stability by adjusting the dihedral angle parameters^30^. This adjustment has a positive effect on the PrP simulation which has a high ratio of helix structure. The particle-mesh Ewald (PME) method was applied for long-range electrostatic interactions^31^, and short-range and non-bonded interactions had a 9 Å distance cutoff. The SHAKE algorithm was used for constraining of the bond length of hydrogen atoms^32^. The temperature was adjusted by Langevin dynamics, with collision frequency (γ) = 2.0^33,34^. We performed the energy minimization (4,000 steps), and the system temperature was then gradually increased to the target temperature before production running. After the heating step, we applied 0.5 kcal/mol restraint on the protein backbone for 1 ns side chain equilibration. The T-REMD production run was performed with 14 replicas (temperature: 300.00, 304.25, 309.00, 313.75, 318.31, 323.00, 327.19, 332.31, 337.19, 342.06, 346.88, 351.81, 356.31, and 360.81)^35^, with a cumulative simulation time of 28 μs (14 replicas and 2μs for each replica) under the NVT ensemble. We used the last 1.5 μs ensemble in each replica to get 2,100 ensembles, and the structural figure was prepared using VMD software^36^.

### Thermodynamic integration (TI)

Using the TI method, we calculated the protein folding free energy using the AMBER18 simulation package with ff14SB force field^30^. We applied a thermodynamic cycle (Fig. S2) wherein ΔG1 (ΔG3) is the free energy difference between the wild-type and mutant PrP at folded (at unfolded) state. ΔG2 (ΔG4) is the free energy difference between folded and unfolded state of wild-type (mutant) PrP. The thermodynamic equality, ΔG1 = ΔG2 + ΔG3 – ΔG4, holds for the thermodynamic cycle. This gives the protein folding free energy difference between the wild-type and mutant PrP, namely ΔΔG = ΔG2 – ΔG4 = ΔG1 – ΔG3. We used the TI method to calculate ΔG1 and ΔG3. The TI calculation uses the mixed potential function, ${H}_{{\rm{\lambda }}}={H}_{wt}+{\rm{\lambda }}({H}_{mut}-{H}_{wt})$, and softcore potentials are used for smooth appearance and disappearance of atoms in vdW and electrostatic interactions in the hybrid state^37^, with α = 0.5, β = 12.0:

$$\begin{array}{ccc}{V}_{wt,disappearing}^{vdW} & = & 4\varepsilon (1-{\rm{\lambda }})[\frac{1}{{[\alpha {\rm{\lambda }}+{(\frac{{r}_{ij}}{\sigma })}^{6}]}^{2}}-\frac{1}{\alpha {\rm{\lambda }}+{(\frac{{r}_{ij}}{\sigma })}^{6}}]\\ {V}_{mutant,appearing}^{vdW} & = & 4\varepsilon {\rm{\lambda }}[\frac{1}{{[\alpha (1-{\rm{\lambda }})+{(\frac{{r}_{ij}}{\sigma })}^{6}]}^{2}}-\frac{1}{\alpha (1-{\rm{\lambda }})+{(\frac{{r}_{ij}}{\sigma })}^{6}}]\\ {V}_{wt,disappearing}^{elec} & = & (1-\,{\rm{\lambda }})\frac{{q}_{i}{q}_{j}}{4\pi \varepsilon \sqrt{\beta {\rm{\lambda }}+{r}_{ij}^{2}}}\\ {V}_{mutant,appearing}^{elec} & = & {\rm{\lambda }}\frac{{q}_{i}{q}_{j}}{4\pi \varepsilon \sqrt{\beta (1-{\rm{\lambda }})+{r}_{ij}^{2}}}\end{array}$$

We computed the free energy difference, ΔG, from the following Gaussian quadrature formulae:

$$\varDelta {\rm{G}}={\int }_{0}^{1}\frac{{\rm{\partial }}G(\lambda )}{{\rm{\partial }}\lambda }d\lambda ={\int }_{0}^{1}{ < \frac{{\rm{\partial }}V(\lambda )}{{\rm{\partial }}\lambda } > }_{\lambda }d\lambda \approx \sum _{i}{w}_{i}{ < \frac{{\rm{\partial }}V(\lambda )}{{\rm{\partial }}\lambda } > }_{i}$$

We used nine point quadrature, setting λ to 0.01592, 0.08198, 0.19331, 0.33787, 0.5, 0.66213, 0.80669, 0.91802, and 0.98408, with weights of 0.04064, 0.09032, 0.13031, 0.15617, 0.16512, 0.15617, 0.13031, 0.09032, and 0.04064, respectively^38^. We performed 0.5 ns TI simulation for each λ value, yielding a cumulative TI simulation time of 2,100 (ensembles) × 9 (λ points) × 2 (protein states, folding and unfolding) × 6 (mutant type) × 0.5 ns = 113.4 μs.

### Defining unfolding states

The unfolding state of PrP cannot be defined using a single structure. In previous studies, unfolding states were replaced by short peptides, which gave reasonable results^39^. From the human PrP sequence, three amino acid sequences were selected before and after the target sequence to make these short peptides (Fig. S3).

## Results

### V180I mutant could destabilize the glycan structure

The subcellular location of PrP is highly dependent on the proteinase K (PK) resistance and aggregation ability^14^. Previous studies on the effect of N-glycosylation on the subcellular localization of PrP reported that the wild-type and monoglycosylated mutant (N181D and N197D) are anchored to the plasma membrane^40^, but the T183A mutant and unglycosylated mutant (N181D/N197D) exist in the cytoplasme^15,40^. In the case of pathological mutant V180I, PrP^V180I^ is equally distributed between the cytoplasm and plasma membrane^40^. Therefore, the subcellular location of PrP is not only linked to the existence of the two glycans, but also the structural stability of PrP.

To predict the change in the stability of glycan in PrP^V180I^, we performed a sequence analysis. We used a well-established dataset for examining the N-glycosylation site. This dataset is based on 2,964 glycoproteins, comprising a list of 11,461 positive and 12,000 negative sequences, with an average length of 41 residues^41^. We sorted out the ASN-ILE-THR (NIT) glycosylation motif from the list of 11,461 positive sequences. As a result, we obtained a list of 480 sequences. An analysis of the position right before the NIT motif (position: −1) revealed that valine is the most frequently observed amino acid (rank: 1), while isoleucine is ranked in the middle (rank: 10) (Fig. S4). In comparison, the frequencies of valine and isoleucine were ranked 5 and 9, respectively, in both positive and negative dataset (Fig. S4). Therefore, valine yields a more stable glycan structure than isoleucine, when it is located before the NIT glycosylation motif. This means that pathologic mutant PrP^V180I^ could destabilize the glycan structure.

### T183A mutant critically destabilizes the hydrophobic core in PrP

**Figure 1:**
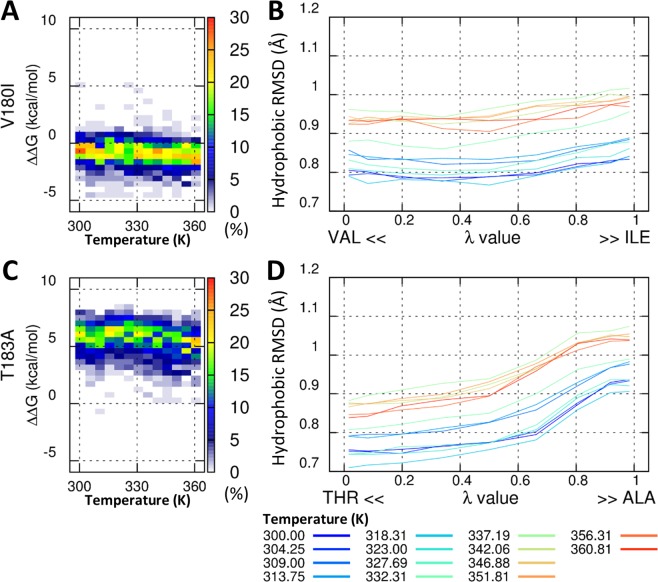
Folding free energy for V180I and T183A mutants. Probability heat map of the folding free energy difference between wild-type and mutant for V180I (**A**) and T183A (**C**) over the indicated temperature range. The packing of the hydrophobic core was measured by core RMSD of six hydrophobic residue positions for V180I (**B**) and T183A (**D**).

We also estimated the mutation-induced changes in protein folding stability. The protein folding free energy difference between wild-type and the mutants was calculated by TI simulation. The ΔΔG value showed no significant change in V180I (<ΔΔG^V180I^ >_T=300K_ = –0.37 ± 0.96 kcal/mol, Fig. 1A), but the T183A mutant was significantly destabilized, with ΔΔG_T=300K_ = 6.25 ± 0.92 kcal/mol (Fig. 1C). The effect of mutation on the packing of hydrophobic core was measured by RMSD of six hydrophobic residue positions (V176, V180, T183, V210, I215, Y218, and hybrid state amino acid). Figure 1B shows no change in the packing of the hydrophobic core in the V180I mutant, when evolving from valine to isoleucine (increasing λ) across the entire temperature range (<ΔRMSD^V180I,T=300K^> = 0.036 Å, Fig. 1B). However, the packing of the hydrophobic core was a little released in the T183A mutant (<ΔRMSD^T183A,T=300K^> = 0.181 Å, Fig. 1D).

### Hydrophobic interactions in PrP^V180I^ and PrP^T183A^

Changes at the atomic details due to the pathological mutants were estimated by calculating the exposure surface of hydrophobic atoms to the solvent. We calculated the reduction in the solvent-accessible surface area (SASA) for each amino acid, caused by the other surrounding amino acids. Therefore, we computed the pair-wise solvent-accessible surface area (SASA) at the amino acid scale. The reduction in SASA for amino acid i by amino acid j was defined by:

$$\begin{array}{ccc}\Delta SAS{A}_{i;j}^{{\rm{\lambda }}} & = & \Delta SAS{A}_{i;j}\,(i\,and\,j\ne mutated\,position)\\ \Delta SAS{A}_{i;j}^{{\rm{\lambda }}} & = & (1-{\rm{\lambda }})\Delta SAS{A}_{i;j}^{wt}+{\rm{\lambda }}\Delta SAS{A}_{i;j}^{mut}\,(i\,or\,j=mutated\,position)\end{array}$$

**Figure 2:**
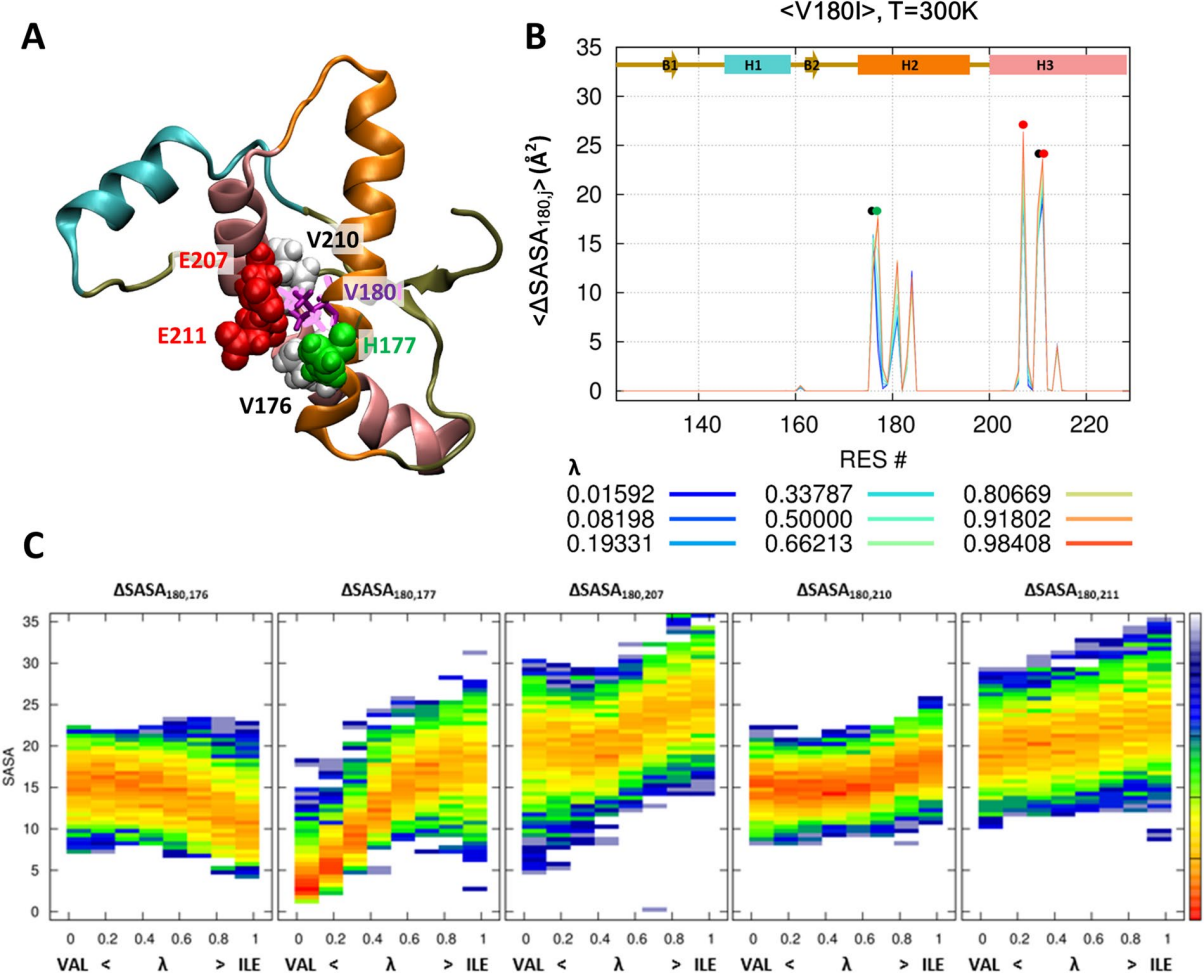
Hydrophobic interaction at the 180^th^ position. The hybrid state of PrP^V180I^ is shown for the 180^th^ position (purple and pink stick) and around the amino acid (ball) (**A**). Reduced solvent-accessible surface area by other amino acids is calculated for the 180^th^ position (**B**). Statistical free energy heat map in thermal energy units (–ln p[ΔSASA]) is plotted as a function of λ for the five amino acids that are in close contact with the 180^th^ position (**C**). Protein structure (**A**) is drawn with VMD version 1.9.1. (http://www.ks.uiuc.edu/Research/vmd/).

**Figure 3:**
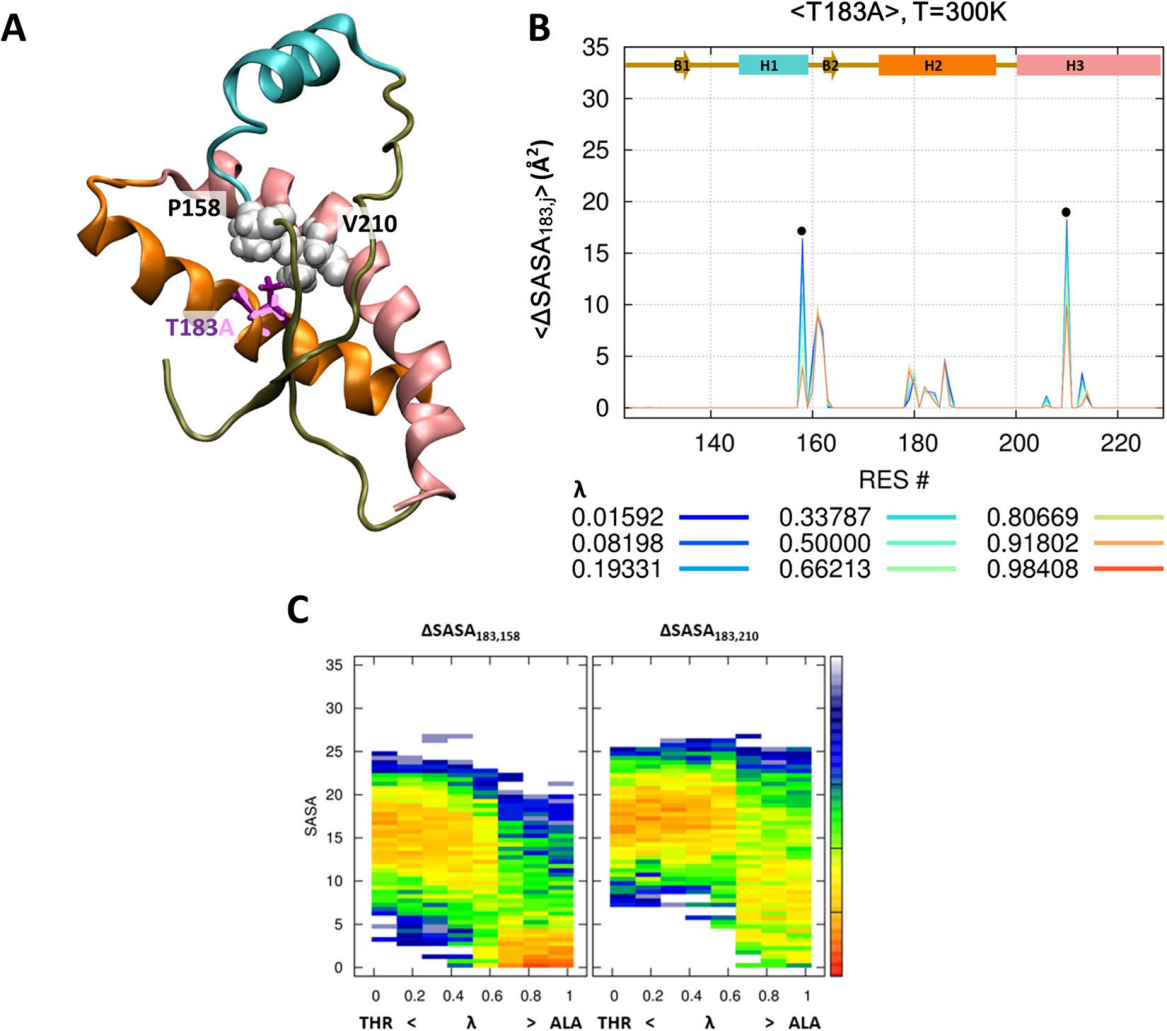
Hydrophobic interaction at the 183^th^ position. The hybrid state of PrP^T183A^ is shown for the 183^th^ position (purple and pink stick) and around the amino acid (ball) (**A**). Reduced solvent-accessible surface area by other amino acids is calculated for the 183^th^ position (**B**). Statistical free energy heat map in thermal energy units (–ln p[ΔSASA]) is plotted as a function of λ for the two amino acids that are in closely contact with the 183^th^ position (**C**). Protein structure (**A**) is drawn with VMD version 1.9.1. (http://www.ks.uiuc.edu/Research/vmd/).

Figures 2A,B show the surface area of the 180^th^ amino acid covered by other amino acids. We chose five amino acids to check the hydrophobic effect on the V180I mutant with a cutoff of 15Å^2^ in Fig. 2B. Valine is extensively covered by the surrounding amino acids, i.e. V176 on H2 and E207, V210, and E211 on H3 (Fig. 2A). We focused on the change in covered surface area during the evolution from valine to isoleucine. Figure 2C shows the statistical free energy heat map for the ΔSASA_i;j_ of specific amino acid pairs over the lambda. The covered surface area increases during the evolution from valine to isoleucine by H177, E207, V210, and E211 (except for the first figure in Fig. 2C). It means that the V180I mutant stabilizes the hydrophobic interaction with H3. In the case of T183A mutant, threonine has a close contact with P158 on H1 and V210 on H3 (Fig. 3A,B). The contact with both amino acids significantly weakened as the λ increased (Fig. 3C). Therefore, the T183A mutation destabilizes the hydrophobic interaction with H1 and H3. In addition, we could identify the irrelevant positions through mutations. There was no effect on V161 and Y162, despite the strong hydrophobic interaction at the 183^th^ position of PrP (Fig. 3B).

V180I, which is the most frequently reported mutation in Japan, has low estimates of penetrance (~1%). Moreover, neurodegenerative diseases caused by V180I occur at an old age (~76.5 years) and progress slowly (~25 month)^42^. Although the subcellular localization of PrP^V180I^ remains changed, our simulation data show that PrP^V180I^ maintains the stable structure, with a reduction in the exposure surface of hydrophobic atoms to the solvent by H177, E207, V210, and E211. This stable structure could act as a factor of low penetrance and slow progress of neurodegenerative diseases. T183A mutation, which breaks the glycosylation motif, not only critically affects membrane anchoring^15^, but also reduces thermostability (temperature of unfolding, ΔT_m_ = 11.4 °C)^43,44^. These experimental data have a good correlation with our simulation data, which showed a dramatic change in P158 and V210, induced by the T183A mutation.

### Loss of hydrophobic interaction in V176G mutant

**Figure 4:**
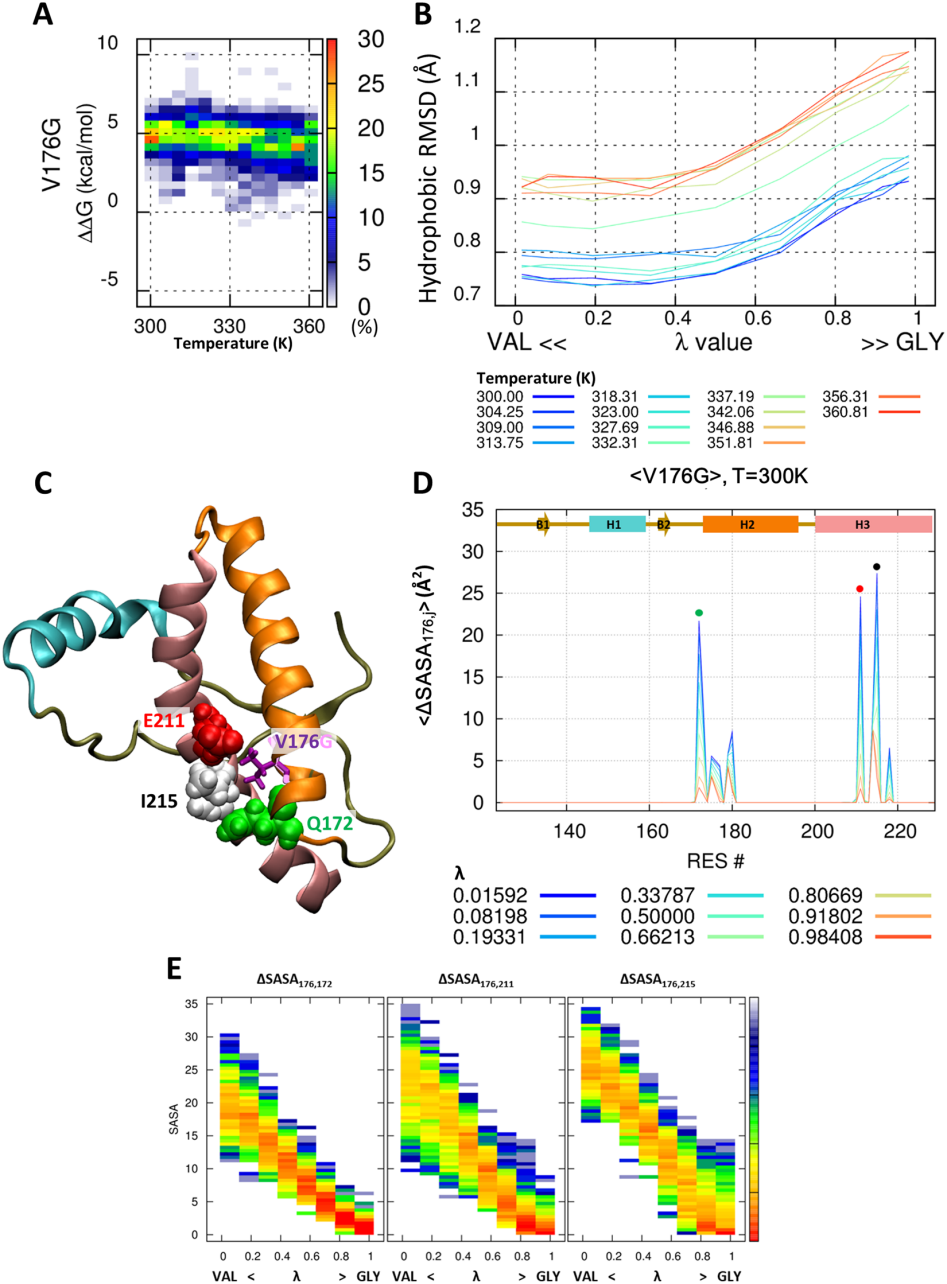
Destabilization of the hydrophobic core by V176G mutant. Probability heat map of the folding free energy difference between wild-type and mutant for V176G over the indicated temperature range (**A**). The packing of the hydrophobic core is measured by core RMSD of six hydrophobic residue positions for V176G (**B**). The hybrid state of PrP^V176G^ is shown for the 176^th^ position (purple and pink stick) and around the amino acid (ball) (**C**). Reduced solvent-accessible surface area by other amino acids is calculated for the 183^th^ position (**D**). Statistical free energy heat map in thermal energy units (–ln p[ΔSASA]) is plotted as a function of λ for the three amino acids that are in closely contact with the 183^th^ position (**E**). Protein structure (**C**) is drawn with VMD version 1.9.1. (http://www.ks.uiuc.edu/Research/vmd/).

Glycine has only one hydrogen atom as a side chain, and therefore, the mutation from valine to glycine results in a loss of hydrophobic interaction with the surrounding amino acids. The ΔΔG reflects the destabilization of PrP^V176G^ (Fig. 4A) and the packing of the hydrophobic core also releases in all temperature (Fig. 4B). Pair-wise ΔSASA showed that all three main hydrophobic interactions were significantly decreased by the glycine mutation (Fig. 4C,D). V176 in wild-type PrP is in close contact with Q172, E211, and I215. These three amino acids are located at H2 (Q172) and H3 (E211 and I215). Distribution of pair-wise ΔSASA of three amino acids is dramatically down to ~0 Å^2^, depending on the λ (Fig. 4E). Our simulation result show that not only hydrophobic residue I215, but also hydrophilic residues Q172 and E211 located around the hydrophobic core, closely interact with V176 and have an important role in stabilization of the hydrophobic core in PrP structure.

### Mutation effect of V210I and the interaction with P158, V180, and T183

**Figure 5:**
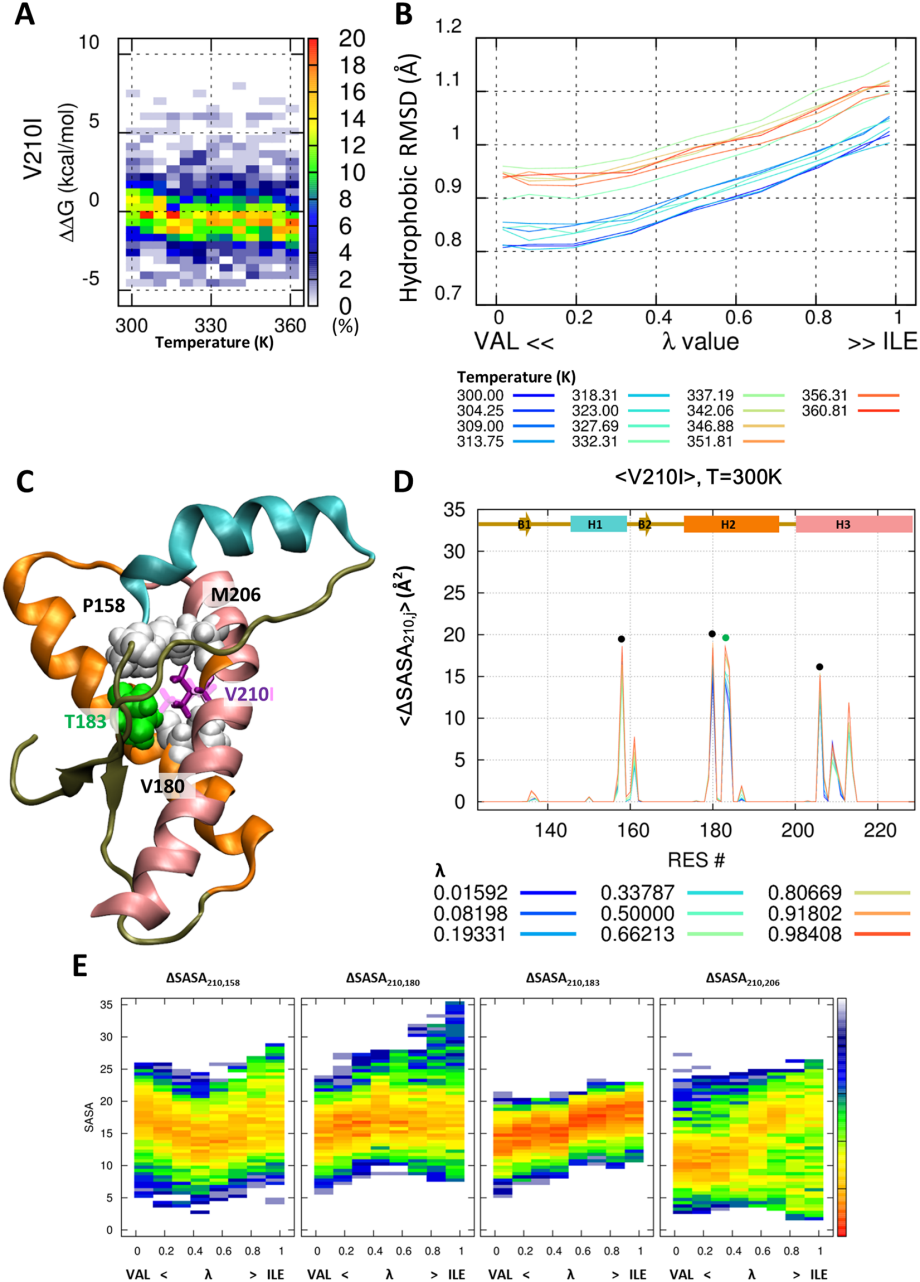
Marginal change in the thermostability by V210I mutation. Probability heat map of the folding free energy difference between wild-type and mutant for V210I over the indicated temperature range (**A**). The packing of the hydrophobic core is measured by core RMSD of six hydrophobic residue positions for V210I (**B**). The hybrid state of PrP^V210I^ is shown for the 210^th^ position (purple and pink stick) and around the amino acid (ball) (**C**). Reduced solvent-accessible surface area by other amino acids is calculated for the 210^th^ position (**D**). Statistical free energy heat map in thermal energy units (–ln p[ΔSASA]) is plotted as a function of λ for the three amino acids that are in close contact with the 210^th^ position (**E**). Protein structure **(C**) is drawn with VMD version 1.9.1. (http://www.ks.uiuc.edu/Research/vmd/).

The V210I mutant is the most commonly reported one in Italy, but it has a low estimated penetrance (only ~10%)^42^. The average age at onset is ~60 years, and duration of progress is ~5 months^42^. Our simulation data showed that the mutation had a marginal effect on structural stability (Fig. 5A). However, the packing of the hydrophobic core becomes weak by mutation from valine to isoleucine (Fig. 5B). Figure 5C,D show that valine is buried by P158 (on H1), Y183, V180 (on H2), and M206 (on H3) at the 210^th^ position. The buried area of these four residues is either not significantly changed by the isoleucine mutation or is slightly increased (Fig. 5E). A previous NMR study on PrP^V210I^ also showed a structural change in the H2-H3 bundle^45^. However, the change in thermal stability of PrP^V210I^ was negligible (ΔT_m_ = 0.1 °C), despite the structural change^46^.

**Figure 6:**
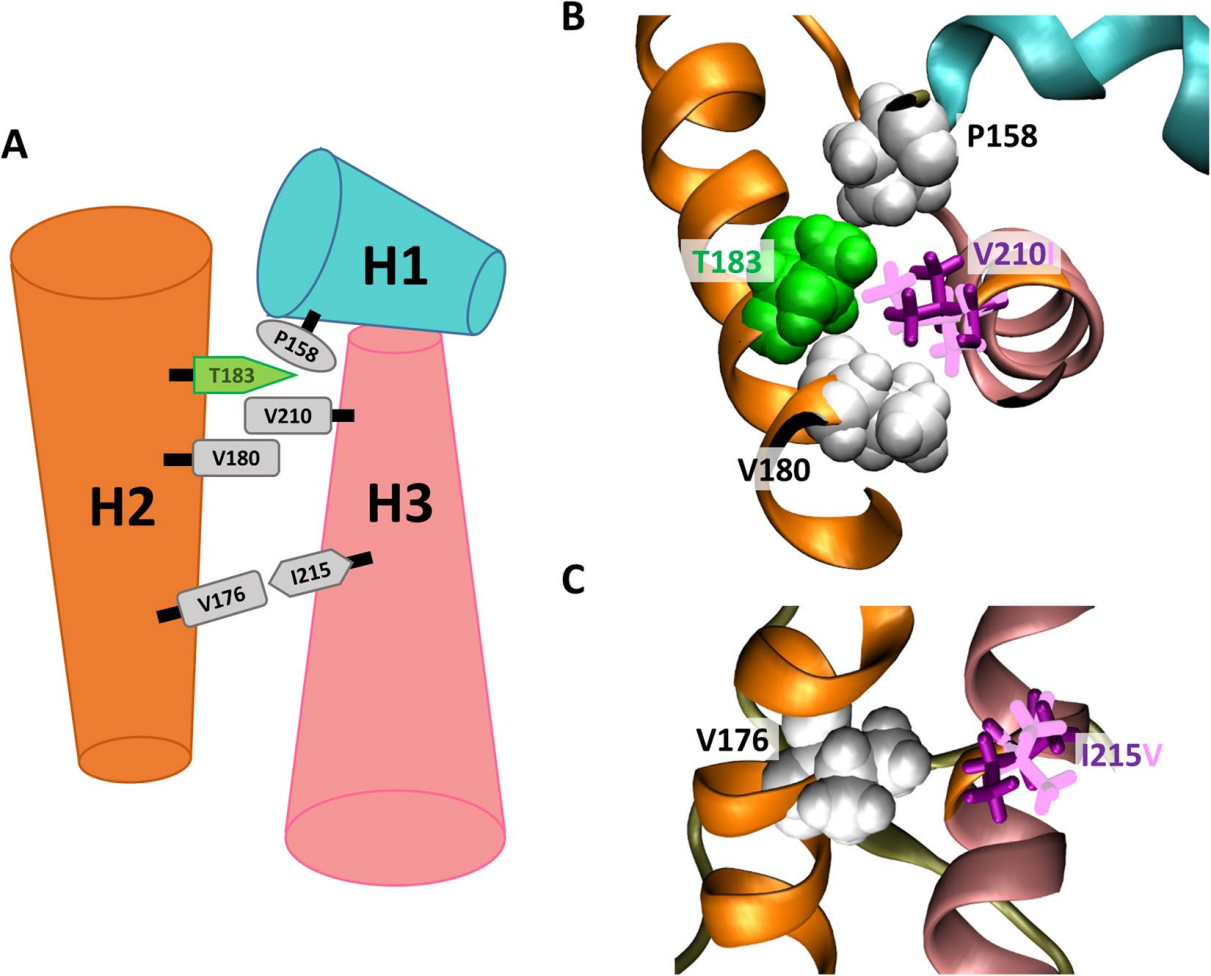
Schematic illustration of PrP. Cartoon model of the three helixes is shown with six amino acids (**A**), in addition to the real structure at atomic level for the hybrid state of PrP^V210I^ (**B**) and PrP^I215V^ (**C**). Protein structures (**B**,**C**) are drawn with VMD version 1.9.1. (http://www.ks.uiuc.edu/Research/vmd/).

The side chain of V210 is located between V180 and T183 at opposite side of the H2-H3 bundle (Fig. 6A,B). V210 and T183 have a strong hydrophobic interaction with P158, which is located in H1 (Fig. 6A,B). Previous studies have shown that the end of H1 is the most fragile within the PrP structure^47^. Moreover, the separation of H1 from H2-H3 bundle greatly affects the thermostability, thereby triggering the conformational change^7^ from PrP^C^ to PrP^SC^. The shape of the side chain in amino acids determines the steric interaction, hydrophobic volume, and buried surface area, which are important to the stability of the hydrophobic core^48^. The mutation from valine to isoleucine results in a slightly increased hydrophobic volume, resulting in a small effect of the V210I mutation on P158. However, the hydrophobic volume is reduced by the mutation from threonine to alanine. The T183A mutation results in the loss of hydrophobic interaction with P158. Owing to this weakening of hydrophobic interaction, H1 is weakly anchored to H2-H3 bundle compared with wild-type PrP. As a result, H1 can be dissociated from the H2-H3 bundle. Previous *in-vitro* and *in-silico* studies show that partially unfolded states have high mobility of H1, and dissociated H1 from H2-H3 bundle is observed before oligomerization^49–52^. The side chain positions of V180 and V210 have similar spatial arrangements on the opposite sides of the hydrophobic core. There is no significant difference between valine-valine (PrP^WT^) and valine-isoleucine (PrP^V180I^ and PrP^V210I^) pair-wise hydrophobic interactions at the 180^th^ and 210^th^ positions. That is one reason why the mutation from valine to isoleucine has a similar effect in V180I and V210I mutants.

### Spatial structures enforce isoleucine at the 215^th^ position

**Figure 7:**
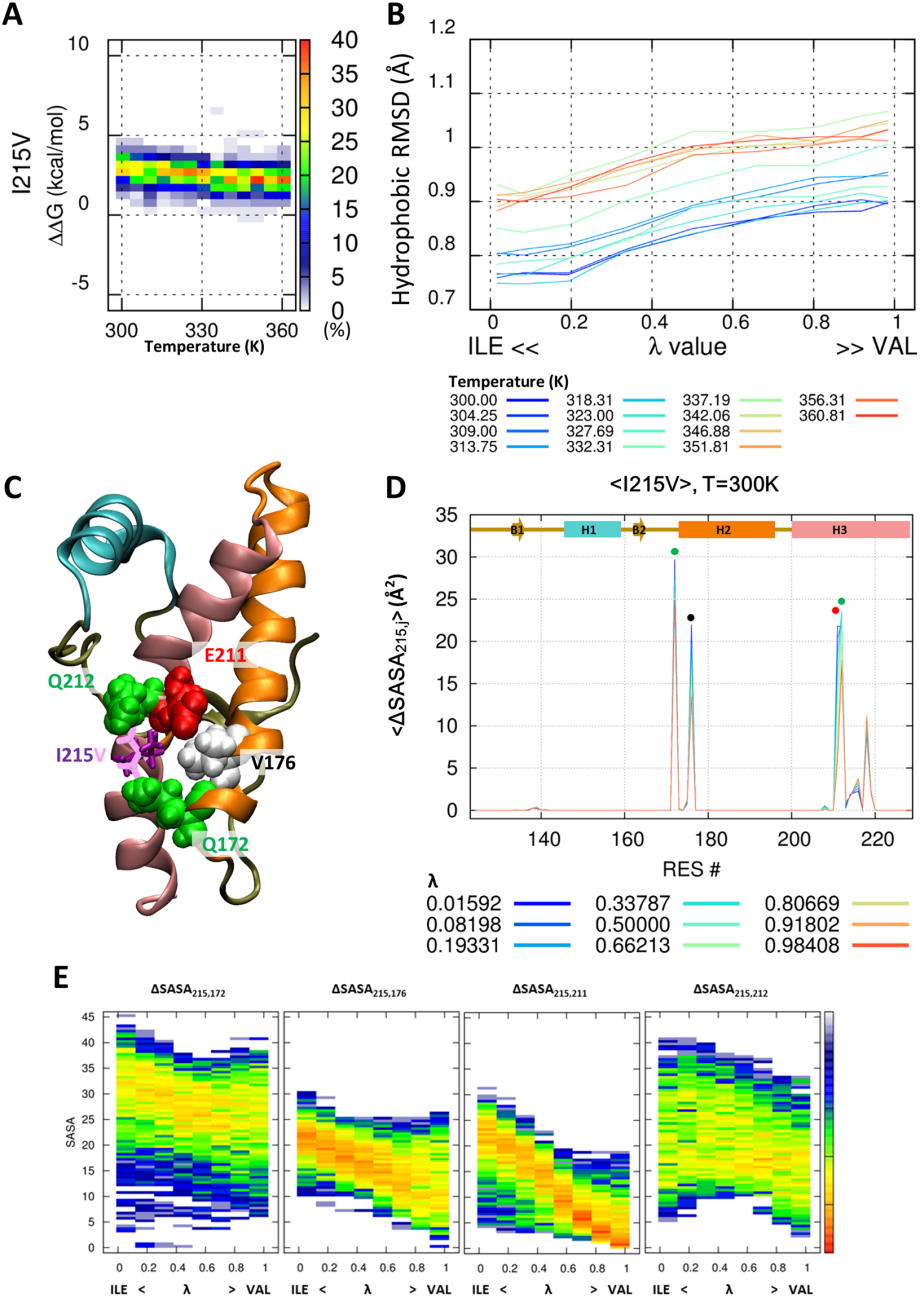
Change in the pair-wise hydrophobic interaction between V176-I215 and V176-V215. Probability heat map of the folding free energy difference between wild-type and mutant for I215V over the indicated temperature range (**A**). The packing of the hydrophobic core is measured by core RMSD of six hydrophobic residue positions for I215V (**B**). The hybrid state of PrP^I215V^ is shown for the 215^th^ position (purple and pink stick) and around the amino acid (ball) (**C**). Reduced solvent-accessible surface area by other amino acids is calculated for the 215^th^ position (**D**). Statistical free energy heat map in thermal energy units (–ln p[ΔSASA]) is plotted as a function of λ for the four amino acids that are in close contact with the 215^th^ position (**E**). Protein structure (**C**) is drawn with VMD version 1.9.1. (http://www.ks.uiuc.edu/Research/vmd/).

We examined the hydrophobic pair-wise interaction of valine-valine (PrP^WT^) and valine-isoleucine (PrP^V180I^ and PrP^V210I^) at the 180^th^ and 210^th^ position. Interestingly, prion has an additional valine-isoleucine pair in the hydrophobic core (Fig. 6A,C). The I215V mutation changes the valine-isoleucine pair to a valine-valine pair. Our simulation result showed that the thermostability of PrP^I125V^ decreased by 3.25 ± 0.65 kcal/mol (Fig. 7A) and the packing of the hydrophobic core was also weakened (Fig. 7B). Figure 7C,D show that isoleucine was in close contact with Q172 and V176 in H2, and E211 and Q212 in H3. The hydrophobic interaction was decreased in four amino acids with increasing λ (Fig. 7E). The notable changes induced by mutation were observed in V176 and E211. E211 and I215 are located in the same helix (H3), so the backbone distance between these two amino acids remained constant. These effects of the mutations arise from the length and direction of the side chain. The length of the side chain decreased a little by the mutation from isoleucine to valine, leading to a weak hydrophobic interaction. For the pairs at the 180^th^ and 210^th^ positions, we showed that there is no significant difference in the hydrophobic interaction between valine-valine and valine-isoleucine. However, the thermostability of PrP is decreased by the change in hydrophobic interaction pair from valine-isoleucine (PrP^WT^) to valine-valine (PrP^I215V^) at the 176^th^ and 215^th^ positions. The main difference between the two pairs is the direction of the interaction surface. The spatial orientation of V176 and I215 interacts with the end of the side chains, and therefore, even a small change in the length of the side chain critically decreases the hydrophobic interaction strength, as observe in the PrP^I215V^ mutant (second figure in Fig. 7E).

### The effect of disappearing of π-stacking in Y218N mutant

**Figure 8:**
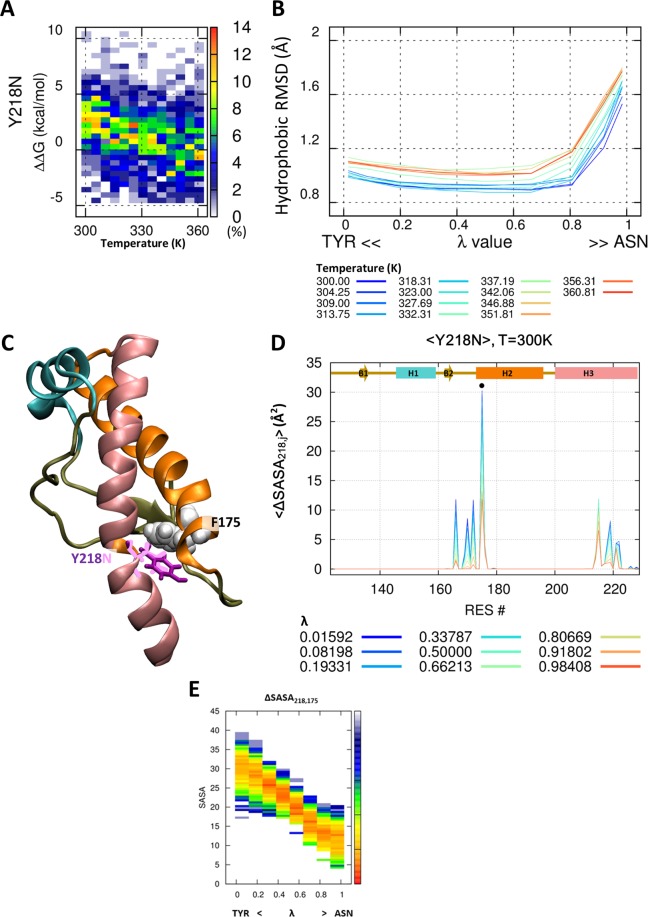
π-stacking between Y218 and F175. Probability heat map of the folding free energy difference between wild-type and mutant for I215V over the indicated temperature range (**A**). The packing of the hydrophobic core is measured by core RMSD of six hydrophobic residue positions for I215V (**B**). The hybrid state of PrP^I215V^ is shown for the 215^th^ position (purple and pink stick) and around the amino acid (ball) (**C**). Reduced solvent-accessible surface area by other amino acids is calculated for the 215^th^ position (**D**). Statistical free energy heat map in thermal energy units (–ln p[ΔSASA]) is plotted as a function of λ for the four amino acids that are in closely contact with the 215^th^ position (**E**). Protein structure (**C**) is drawn with VMD version 1.9.1. (http://www.ks.uiuc.edu/Research/vmd/).

The PrP^Y218N^ mutant has the most fluctuating hydrophobic core structure in the six mutants. The mutation from tyrosine to asparagine induces a dramatic decrease in the hydrophobicity at all temperatures, <ΔRMSD^Y218N, T=300K^ > = 0.536 Å (Fig. 8B), and fluctuations in the hydrophobic core yield a broad range of ΔΔG (Fig. 8A). Stability of PrP^Y218N^ was also decreased by 3.27 ± 2.58 kcal/mol at T = 300 K, with the disappearing of the aromatic ring in the tyrosine. Tyrosine at the 218^th^ position is in close contact with F175 in PrP^wild^. Figure 8C and 8D show the attractive nonbonded interaction known as π-stacking between the aromatic rings of tyrosine and phenylalanine. The favorable distance and orientation of the aromatic rings in Y218 and F175 enforce a specific spatial configuration of PrP^wild^. However, ΔSASA is significantly decreased by the mutation from tyrosine to asparagine, which result in a loss of the aromatic ring (Fig. 8E). Interestingly, a previous circular dichroism (CD) spectroscopy study with F175A mutant showed the importance of this π-stacking interaction, in the form of a significant reduction in the melting temperature^53^ (ΔT_m, F175A_ = 8 °C).

## Discussion

The hydrophobic core has a crucial function to maintain the normal structure and prevent aggregation in many protein systems. Mutations in the hydrophobic core disrupt the folding path and expose hydrophobic amino acids to the solvent, leading to misfolding and aggregation of the protein^54,55^. PrP is one of the most common proteins that cause neurodegenerative diseases such as Creutzfeldt-Jakob disease by misfolding and aggregation. It has a hydrophobic core in the middle of H2-H3 bundle, and many pathological mutants related to this hydrophobic core have been reported. To predict the effect of pathogenic mutants, many computational methods have been developed, but their performance is limited by the protein structure sampling and accuracy of the energy function. Therefore, we used T-REMD, a modern molecular dynamics simulation method, for extensive structure ensemble sampling across the energy barrier with 28 μs simulation time, and obtained 2,100 PrP structures. On these ensembles, we strictly applied atomic-scale force field AMBER14SB with TI calculation, which is one of the most popular computational methods to compare the free energy difference between two given states (such as wild-type and mutant proteins). We verify our ΔΔG from TI calculation using 15 different well-known computational methods: FoldX, PoPMuSiC v3.1^56^, CUPSAT^57^, Mupro^58^, mCSM^59^, SDM^60^, DUET^61^, I-mutant2.0(PDB)^62^, I-mutant2.0(sequence)^62^, AUTO-MUTE(SVM)^63^, AUTO-MUTE(RF)^63^, iStable^64^, INPS^65^, EASE-MM^66^, and MAESTRO^67^. These methods showed similar trends as our results, except CUPSAT and SDM. CUPSAT significantly underestimated the effect of V176G mutant, and SDM showed a dramatic stability for the T183A mutant (Fig. S5).

In this study, we examined the first glycosylation motif, which determines the location of PrP in the cell. Many previous *in-vitro* and *in-silico* studies have shown the importance of PrP location in misfolding and aggregation^40,68,69^. The known mechanism for this is that the oligomerization sites are buried in the membrane surface, which prevents the oligomerization and propagation^69^ of PrP^SC^. The T183A mutant not only breaks the first glycosylation motif but also destabilizes the PrP^T183A^ structure. Our results showed good agreement with the findings of previous CD and hydrogen-deuterium exchange coupled with mass spectrometry (HDX-MS) studies. In CD results, thermodynamic stability of the T183A mutant is dramatically decreased by ΔT_m_ = 11.4 °C compared to the wild-type. Our simulation result also shows destabilized PrP^T183A^ structure with <ΔΔG^T183A^> T = 300 = 6.25 ± 0.92 kcal/mol. Destabilized H1 and H3 regions in the PrP^T183A^ are observed in HDX-MS experiments^43^. Interestingly, 183^th^ amino acid is more weakly contacted with P158 on H1 and V210 on H3 by the T183A mutation in our simulation (Fig. 3). However, the V180I mutant no effect on PrP^V180I^ stability. Sequence analysis showed that the mutation from valine to isoleucine at the 180^th^ position could decrease glycan stability. The changed sequence could affect glycan attachment frequency and/or structural fluctuation of glycoprotein PrP^V180I^ on the H2 region. Two glycans significantly stabilize the membrane binding structure of PrP^40^. The loss of first glycan and fluctuation of H2 region destabilize membrane binding structure of PrP. It leads to detachment of PrP^V180I^ from plasma membrane. our sequence analysis provides a clue to the detachment of PrPV180I from the plasma membrane, as observed in cell imaging and western blotting experiments^40^.

We not only calculate the protein stability, but also show the pair-wise amino acid interactions that affect the protein stability at atomic scale. We calculated the pair-wise ΔSASA during the evolution from wild-type (λ = 0) to mutant (λ = 1) protein. The pair-wise ΔSASA provides information on which interaction pairs of amino acids are maintained or weakened. In particular, we compared the hydrophobic pair-wise interaction between valine-valine and valine-isoleucine at the 180^th^–210^th^ and 176^th^–215^th^ positions. These two pairs show a little spatial difference in the direction and orientation of the amino acid side chain (Fig. 6). The I215V mutant, which induces the change from Val176-Ile215 to the Val176-Val215 pair, showed only a decrease in the pair-wise hydrophobic interaction. The V180I (and V210I) mutant, which induces the change form Val180-Val210 to Ile180-Val210 (Val180-Ile210) pair, showed no significant difference in the pair-wise hydrophobic interaction. These results imply that the hydrophobic core has more favorable steric interactions with amino acids at the 176^th^–215^th^ position more than at the 180^th^–210^th^ position, and therefore, the I215V mutant decreases the PrP stability by 3.25 ± 0.65 kcal/mol. In addition, the π-stacking between Y218 and F175 enforces a specific spatial configuration of PrP^wild^. In the Y218N mutant, our simulation results showed that the breaking of π-stacking significantly disrupts the packing of the hydrophobic core and reduces PrP^Y218N^ stability. Previous NMR solution structure and CD spectroscopy data also show the importance of this π-stacking^53^. The F175A mutant, which removes the aromatic ring of F175, induced a direction change in the Y218 within the NMR structure, and a dramatic decrease in the melting temperature (ΔT_m, F175A_ = 8 °C) in CD spectroscopy data^53^.

A full atomic model of PrP^SC^ has been recently reported^70^. They used mouse prion protein to propose 4-rang β-solenoid (4RβS) model. The hydrophobic core must be broken to make the 4RβS structure in this model, so the stability of hydrophobic core is important in 4RβS model. Our studies also said about the importance of hydrophobic core and shows the variation of the stability of the hydrophobic core by six mutants. It helps us to understand the structure conversion of PrPC into PrPSC with 4RβS model, and is also expected to be helped to studies that prevent fibril formation^71^.

## Supplementary information

Supplementary information

Supplementary information

Supplementary information

Supplementary information

Supplementary information
