# Supplementary figures and images for: Atomic insights into the effects of pathological mutants through the disruption of hydrophobic core in the prion protein
Source: Sci Rep. 2019 Dec 16;9:19144. doi: 10.1038/s41598-019-55661-2 (PMC6915724; doi:10.1038/s41598-019-55661-2)

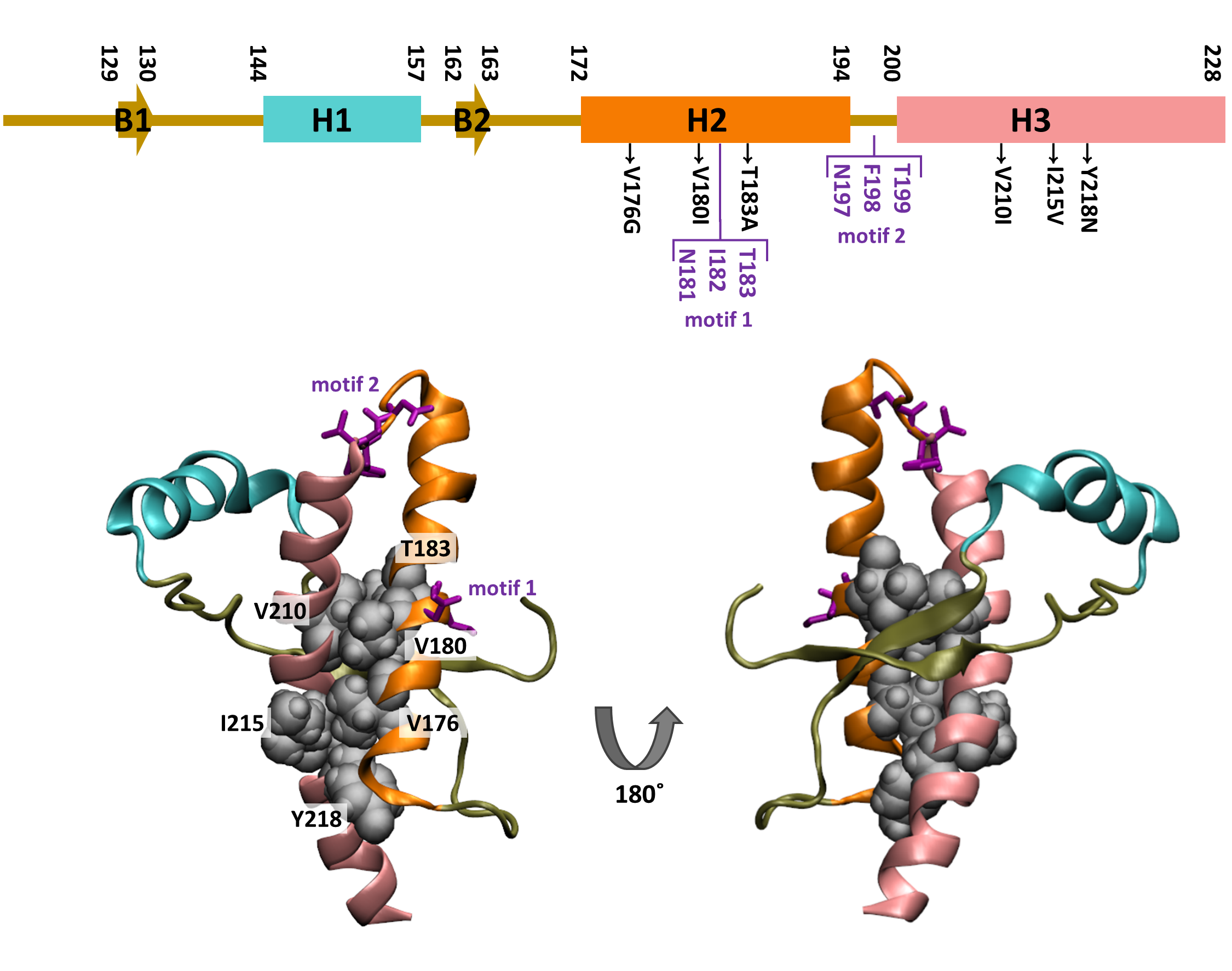

Supplement: Supplementary file 1 — Supplementary information [file 41598_2019_55661_MOESM1_ESM.tif]

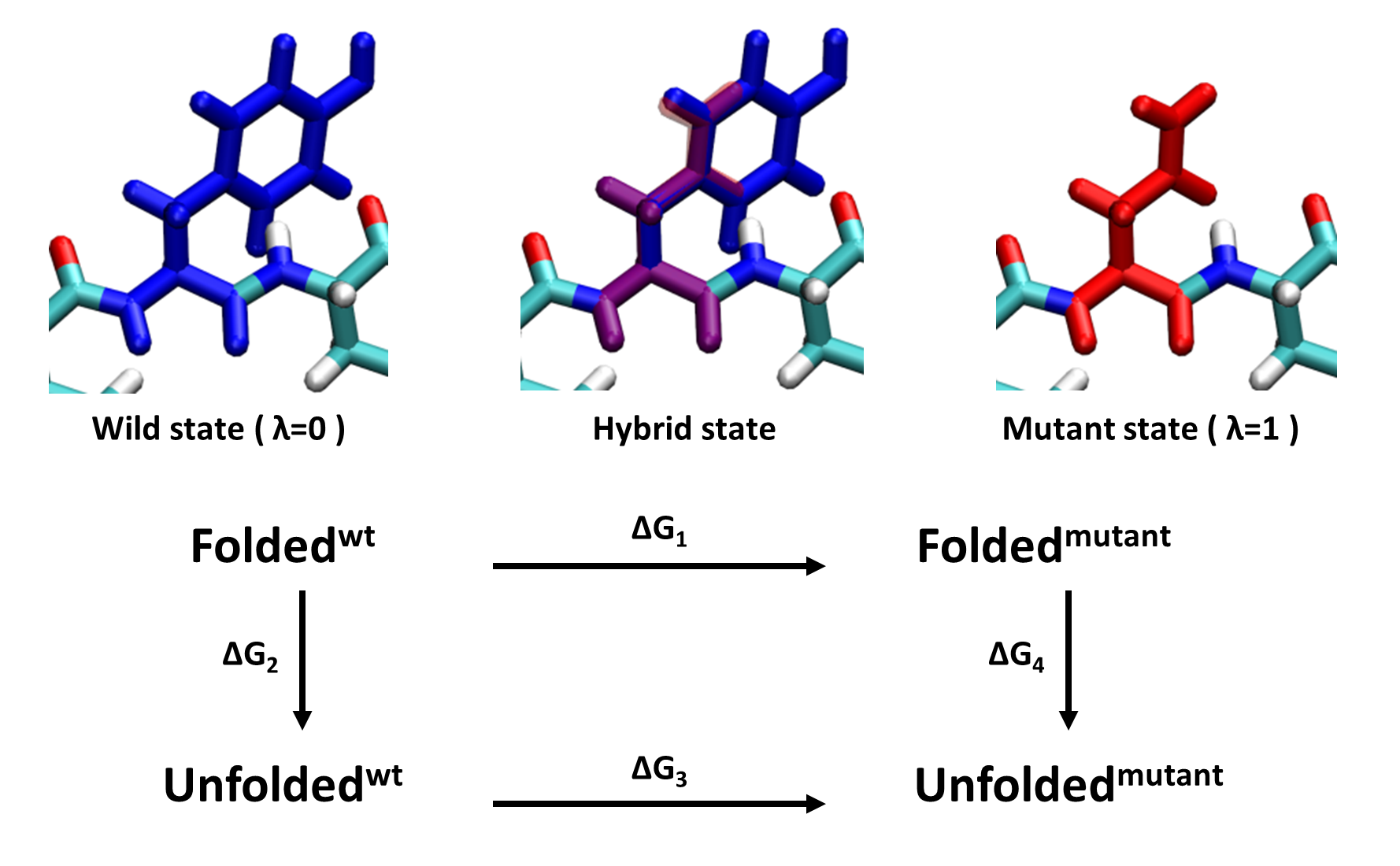

Supplement: Supplementary file 2 — Supplementary information [file 41598_2019_55661_MOESM2_ESM.tif]

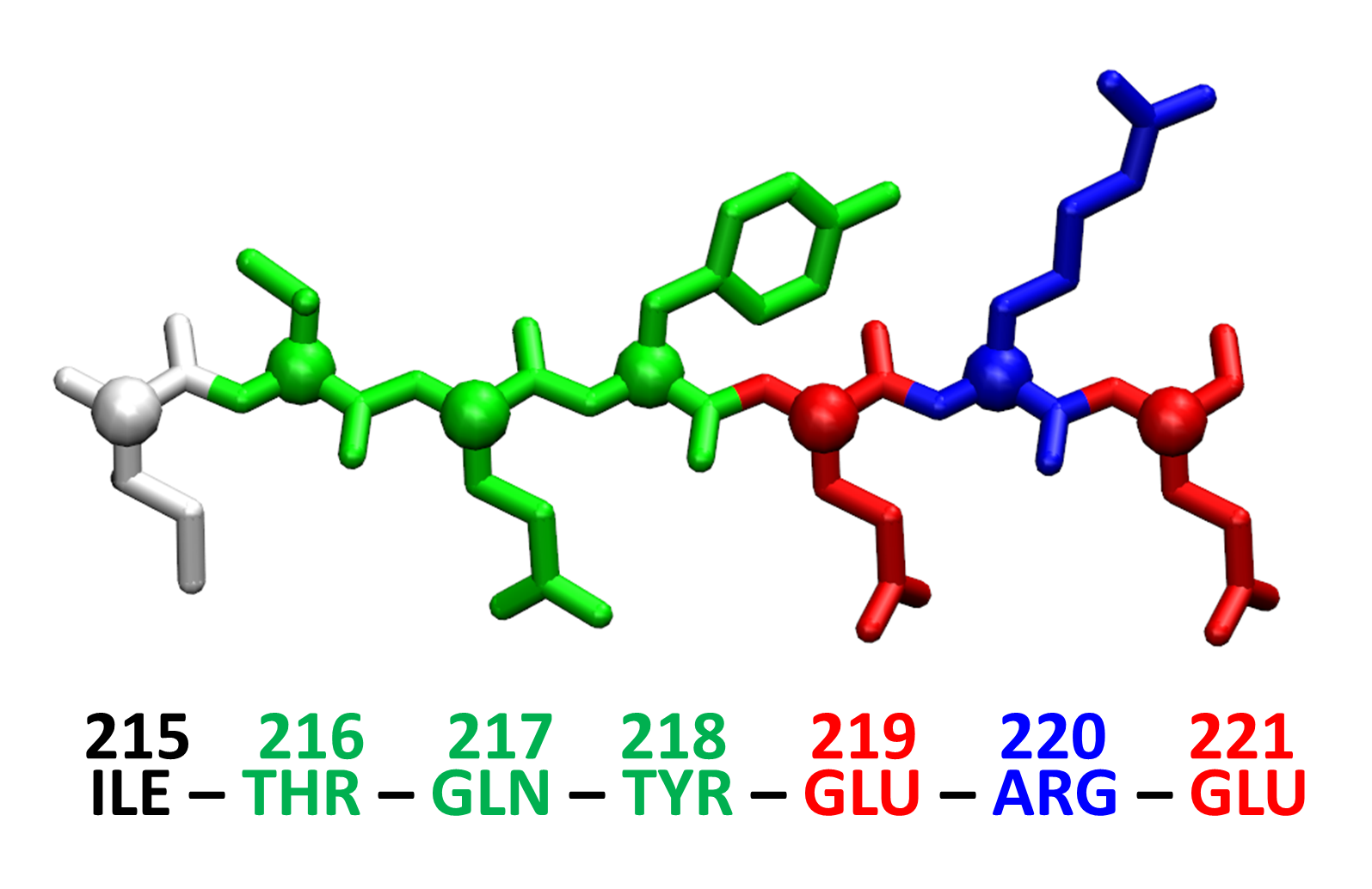

Supplement: Supplementary file 3 — Supplementary information [file 41598_2019_55661_MOESM3_ESM.tif]

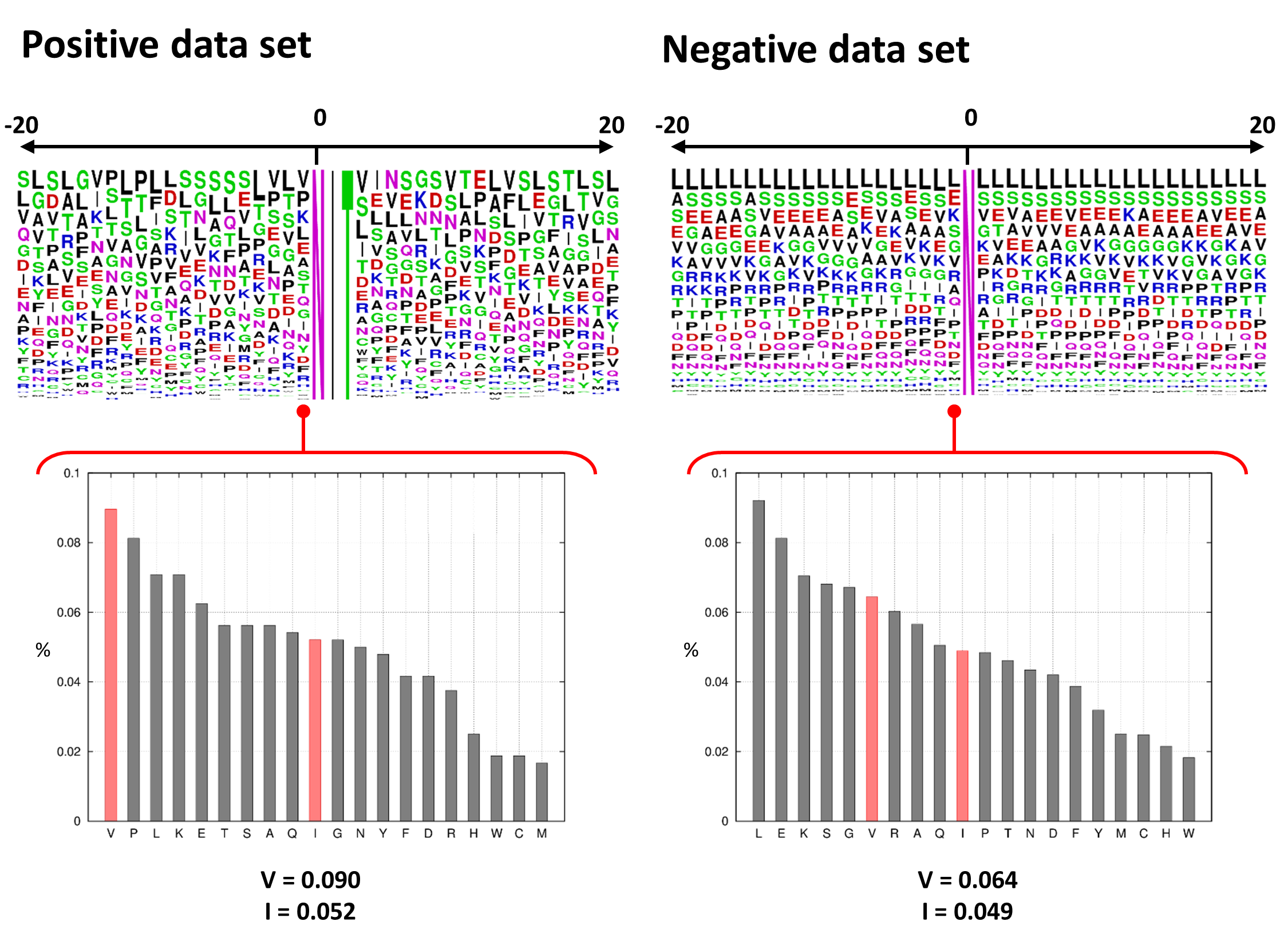

Supplement: Supplementary file 4 — Supplementary information [file 41598_2019_55661_MOESM4_ESM.tif]

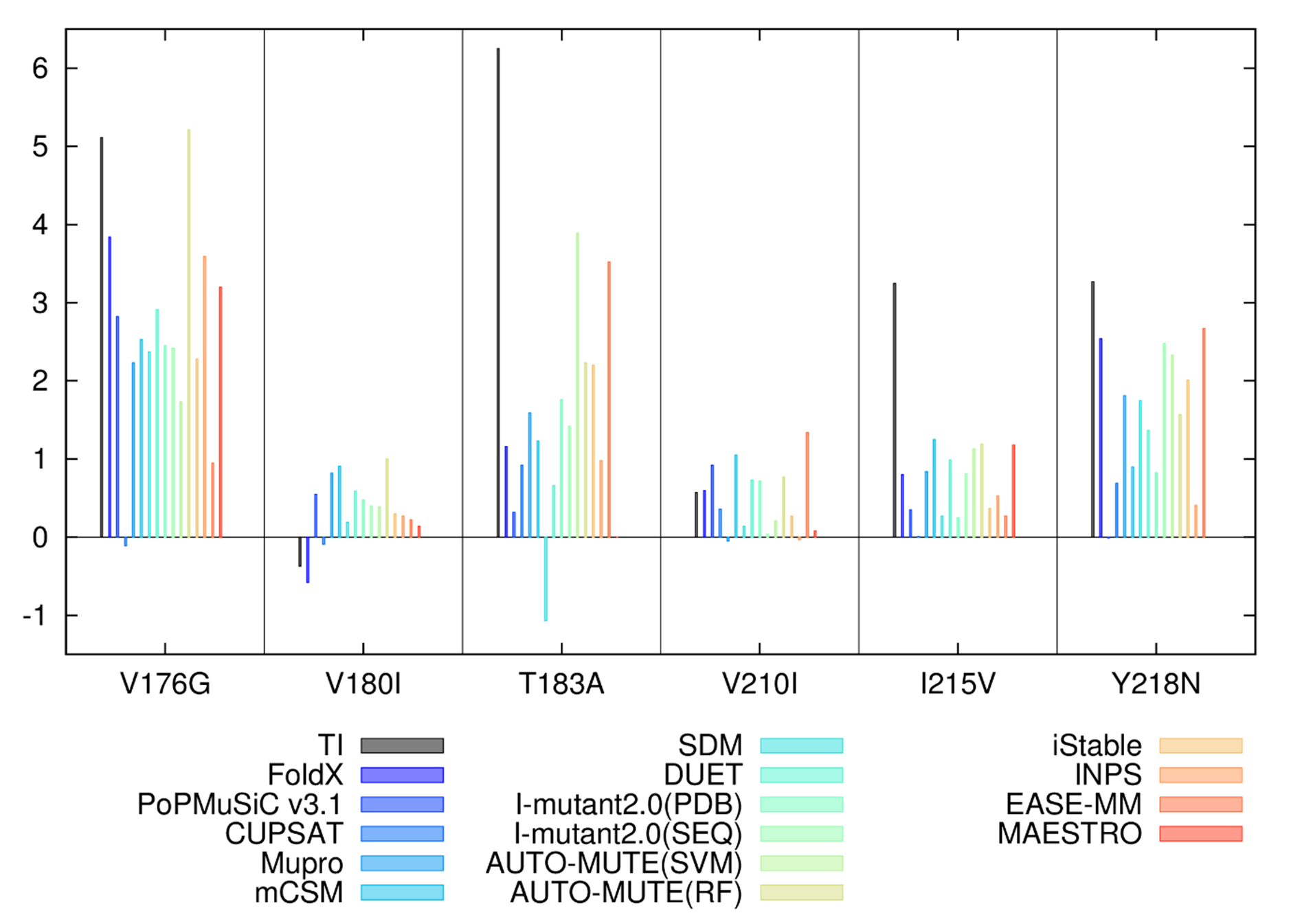

Supplement: Supplementary file 5 — Supplementary information [file 41598_2019_55661_MOESM5_ESM.tif]
